# Predictive factors of perinatal depression among women with gestational diabetes mellitus in the UAE: a cross-sectional clinical study

**DOI:** 10.1186/s12884-024-06307-3

**Published:** 2024-02-19

**Authors:** Khadija I. Alzarooni, Salah Abusnana, Hala Zakaria, Amal Hussein, Bashair M. Mussa, Ghada Mohammed

**Affiliations:** 1 Clinical Sciences Department, College of Medicine, University of Sharjah, 27272 Sharjah, United Arab Emirates; 2 Family Medicine Department, University Hospital Sharjah, Sharjah, United Arab Emirates; 3 Diabetes and Endocrinology Department, University Hospital Sharjah, Sharjah, United Arab Emirates

**Keywords:** Gestational Diabetes Mellitus (GDM), Perinatal Depression (PND), United Arab Emirates (UAE), Edinburgh Postnatal Depression Scale (EPDS)

## Abstract

**Background:**

Gestational Diabetes Mellitus (GDM) is responsible for the development of 30–50% of type 2 diabetes mellitus that predisposes later to adverse consequences among affected mothers and their offspring. Several studies have suggested that GDM increases the risk of developing perinatal depression (PND); however, factors that are involved in this association are yet to be determined. This study aims to identify factors that interrelate GDM and PND among pregnant and postnatal women in the United Arab Emirates (UAE).

**Methods:**

A total of 186 women between 18 and 45 years old attending the obstetrics clinic during their 3rd trimester or up to 6 months postnatal were recruited between October 2021 and April 2022. Women who were known to have pre-existing diabetes mellitus (type 1 or type 2), kidney disease, liver disease, and those receiving hormonal therapy were excluded. Participants completed a structured questionnaire including sociodemographic data and the Edinburgh Postnatal Depression Scale (EPDS). Based on their EPDS scores, study participants were categorized into three groups: no depression (> 9), possible depression (9–11), and high possibility/strong positive depression (≥ 12). SPSS 26 was used for data analysis.

**Results:**

Among the 186 participants, 81% (*n* = 151) were Emirati, 41% (*n* = 76) had no GDM, and 58% (*n* = 110) had GDM. Of the study participants, 34.4% had a high possibility of strong positive depression, 40.9% had possible depression, and only 6.5% had no depression. The association between GDM and PND was clinically and statistically insignificant, with a calculated odds ratio (OR) of 1.574 (*p* value = 0.204) and a 95% confidence interval (0.781—3.172). However, age, personal history of depression, and BMI were found to be strong predictors of depression among pregnant/postpartum women in the UAE.

**Conclusions:**

The study findings propose that age, personal history of depression, and obesity are strong predictors of depression during pregnancy. The strong correlation between obesity (which is a known strong predictor of GDM) and PND suggests that further studies with longitudinal designs and longer observational periods might better reveal the relationship between GDM and PND.

**Trial registration:**

Retrospectively registered study by Research Ethics Committees of the University Hospital Sharjah and the University of Sharjah (Ref. No.: UHS-HERC- 025–17122019) December 17, 2019.

## Background

Gestational Diabetes Mellitus (GDM), is defined as diabetes diagnosed in the 2nd or 3rd trimester of pregnancy that was not clearly overt diabetes prior to gestation [1]. It is reported to have a prevalence of 15 to 18% among pregnant women worldwide [2, 3]. Recent reports estimated that the status of impaired maternal blood glucose levels reached up to 36% [3].

Recently, published reports have shown that during the last two decades, GDM prevalence increased by 10 to 100% among several ethnic groups [4]. Remarkably, GDM is responsible for the development of 30 to 50% of type 2 diabetes mellitus (T2DM) in any population, suggesting that pregnancy and the associated metabolic stress unmask genetic susceptibility to T2DM [5]. The risk factors for GDM include older maternal age, family history of diabetes, and obesity [5].

Another challenge for pregnant women is perinatal depression (PND), which is the most common psychological complication that occurs during pregnancy and up to 12 months after giving birth [6, 7]. PND affects approximately 10 to 15% of women and is manifested by depressed mood or mood swings, excessive emotional reactions, insomnia, anxiety, and panic attacks [8]. In June 2023, the American College of Obstetricians and Gynecologists (ACOG) issued updated recommendations, advising the screening of depression on at least two occasions during pregnancy and once more during a postpartum visit, utilizing validated tools. The Edinburgh Postnatal Depression Screen featuring 10 questions (EPDS) is recommended as one of the two most commonly employed tools [9]. Although PND is associated with impactful outcomes on maternal quality of life and offspring early development, this disorder is understudied, and hence, there are significant gaps in understanding its pathogenesis [10].

A large cross-sectional study conducted in 2018 evaluated factors pertaining to sociodemographic variables, blood pressure (BP) measures, body mass index (BMI), and oral glucose tolerance test (OGTT) levels and their correlation with both depression and GDM. A group of 347 antenatal women with gestational age above 24 weeks were included, and the “Clinically Useful Depression Outcome Scale” (CUDOS) questionnaire was applied for depression assessment. The prevalence of depression among women with GDM was estimated at 56.1% compared to 38.5% among women with no GDM. In addition, women with GDM aged above 30 years, a BMI above 27 kg/m^2^, and middle/high socioeconomic status were found to have an increased risk of depression during pregnancy [11]. Therefore, most literature suggests that the mean age to develop depression is among women in their 30s regardless of their socioeconomic status and educational accomplishment predictions. Thus, pregnant women beyond the age of 30 years may need more psychological evaluation and medical attention if a PND diagnosis is suspected.

Several findings have suggested that GDM increases the risk of developing PND and that there is an association between these disorders [12, 13]. However, the strength and the factors that are involved in this association are yet to be determined. Investigation of common factors between GDM and PND, such as hyperglycemia, insulin resistance, and oxidative stress, has led to inconsistent and limited outcomes [13].

The present study aims to identify the factors, if any, that interrelate GDM and PND among pregnant and postnatal women in the UAE.

## Methods

### Study aim

This study aims to identify factors that interrelate GDM and PND among pregnant and postnatal women in the United Arab Emirates (UAE).

### Study design

This is a cross-sectional study where pregnant women and postnatal women up to 6 months post-delivery were recruited from maternity outpatient clinics located at a semi-government hospital in the UAE. All data pertaining to sociodemographic characteristics and clinical variables were collected at a single point in time.

### Ethical approval

This study was approved by the Research Ethics Committees of the University Hospital Sharjah (UHS) and the University of Sharjah (UOS) (Ref. No.: UHS-HERC- 025–17122019). Informed consent forms were signed by study participants before their enrollment in the study. This research was conducted in alignment with the ethical principles of the Declaration of Helsinki.

### Inclusion and exclusion criteria

The study included pregnant and postnatal women (up to 6 months post-delivery) between 18 and 45 years of age. Women with pre-existing diabetes mellitus (type1 or type), kidney disease, liver disease, and those receiving hormonal therapy were excluded from the study. Sample size calculation was done, using the formula *n* = [Z_1-α/2_ + Z_1-β_]^2^ *[{p_1_*(1-p_1_) + p_2_*(1-p_2_)}/(p_1_-p_2_)^2^], for detecting a difference between two proportions: proportion of PND in women with GDM (p_1_) compared to that in women with no GDM(p_2_) For the estimated proportions, p_1_ and p_2_, of 56.1% and 38.5% [11], a 95% confidence level, and study power of 80%, the minimum sample size needed to conduct this study was 244 (122 women per each group).

### Data collection

Women who fit the study’s eligibility criteria were invited to participate in the study. After signing informed consent forms, study participants were asked to fill out a questionnaire that inquired about their demographics, including their age, nationality, marital status, and socioeconomic status. A detailed medical history related to their pregnancy was also collected. The questionnaire also asked about a woman’s personal history of depression as well as depressive symptoms experienced during or after pregnancy. The occurrence of PND was assessed using the Edinburgh Postnatal Depression Scale (EPDS), a widely used screening tool designed to assess and identify symptoms of depression in women during the perinatal period, which includes both pregnancy and the postpartum period [14]. The EPDS It includes questions that address both antenatal (before birth) and postnatal (after birth) aspects of maternal mental health. In addition, participants’ vital signs, weight, and height were measured, and their BMI values were calculated accordingly. GDM was diagnosed based on the results of the OGTT test, which is a routine screening test performed for any pregnant woman between 24 and 28 weeks of gestation, with no known prior history of diabetes. The OGTT test results were retrieved from the medical records of the study participants.

### Data analysis

Data were analyzed using SPSS software, version 26.0 (SPSS, Chicago, IL, USA). Normally distributed continuous data such as age and depression score were expressed as the means and standard deviations (SD), whereas skewed data were summarized and reported using medians and interquartile ranges. Categorical data such as GDM diagnosis, nationality, socioeconomic status, BMI, pregnancy information, and depression groups were expressed as counts and percentages. The EPDS score, which was used to measure depression status in participants, was calculated for each participant based on her answers to the 10-question EPDS scale. EPDS scores ranged between 0 and 30, where a score less than 9 indicated “no depression”, a score of 9 to 12 indicated “possible depression” and a score above 12 indicated "high possibility/strong positive depression". The chi-square test was used to measure the association between categorical variables, and the odds ratio (OR) was reported to reflect the strength of an association. A multivariate binary logistic regression model was used to predict “High possibility/Strong positive depression” using the input variables that showed statistical significance in the bivariate analysis. The enter method was used to conduct the regression analysis. The Mahalanobis distance was used to check for the presence of multivariate outliers. The Omnibus test was used to assess the significance of the regression model, while the Hosmer and Lemeshow test was conducted to evaluate the adequacy of the data. *P* values below 0.05 were considered statistically significant.

## Results

### Sociodemographic information

A total of 186 participants were recruited for the study and completed the questionnaire. The mean age of the sample was 30.54 years (SD = 5.58), 81.2% (*n* = 151) were Emirati, 98.9% (*n* = 184) were married and 95.7% (*n* = 178) had middle socioeconomic status. Forty-three percent (*n* = 80) of the study participants were obese, and 35.5% (*n* = 66) were overweight. Table 1 summarizes the sociodemographic characteristics of the participants.

**Table 1:** Sociodemographic characteristics of participants (*n* = 186)

*Variables*	*Mean* ± *SD*	
***Age (years)***	30.54 ± 5.58	
	***N***	***%***
***Age groups***		
*18–25*	42	22.6
*26–35*	105	56.5
*36–45*	39	21.0
***Nationality***		
*Emirati*	151	81.2
*Arab expatriate*	19	10.2
*Non-Arab expatriate (Asian)*	14	7.5
*Non-Arab expatriate (Western nationality)*	2	1.0
***Marital status***		
*Married*	184	98.9
*Separated*	2	1.1
***Socioeconomic status***		
*Low*	2	1.1
*Middle*	178	95.7
*High*	6	3.2
***BMI***		
*Underweight/Normal*	40	21.5
*Overweight*	66	35.5
*Obese*	80	43.0

### Depression and Edinburgh Postnatal Depression Scale (EPDS) univariate analysis

Table 2 presents the descriptive analysis of the EPDS scale items among the recruited participants (*n* = 186). 21.5%, *n* = 40) *have been unable to laugh and see the funny side of things*, (25.8%, *n* = 40) *have not looked forward with enjoyment to things*, (18.3%, *n* = 34) *have blamed themselves unnecessarily when things went wrong*, (57%, *n* = 106) *have been anxious or worried for no good reason*, (72%, *n* = 134) *have felt scared or panicky for no very good reason*, (14.5%, *n* = 27) have been felt that *things have been getting on top of them*, (69.9%, *n* = 130) *have been so unhappy that have had difficulty sleeping*, (12.9%, *n* = 24) *have felt sad or miserable*, (61.3%, *n* = 114) *have been so unhappy that have been crying* and (1.1%, *n* = 2) *have thought of harming self* which in this step warrants an urgent medical attention for child and mother safety.

**Table 2:** Edinburgh Depression Scale item analysis (*n* = 186)

*Variables*	*N*	*%*
***1. I have been able to laugh and see the funny side of things***		
*As much as I always could*	146	78.5
*Not quite so much now*	28	15.1
*Definitely not so much now*	9	4.8
*Not at all*	3	1.6
***2. I have looked forward with enjoyment to things***		
*As much as I ever did*	138	74.2
*Rather less than I used to*	28	18.3
*Definitely less than I used to*	9	5.9
*Hardly at all*	3	1.6
***3. I have blamed myself unnecessarily when things went wrong***		
*Yes, most of the time*	7	3.8
*Yes, some of the time*	27	14.5
*Not very often*	92	49.5
*No, never*	60	32.3
***4. I Have been anxious or worried for no good reason***		
*No not at all*	64	34.4
*Hardly ever*	16	8.6
*Yes, sometimes*	83	44.6
*Yes, very often*	23	12.4
***5. I have felt scared or panicky for no very good reason***		
*Yes, quite a lot*	48	25.8
*Yes, sometimes*	86	46.2
*No, not much*	34	18.3
*No, not at all*	18	9.7
***6. Things have been getting on top of me***		
*Yes, most of the time I haven’t been able to cope at all*	6	3.2
*Yes, sometimes I haven’t been coping as well as usual*	21	11.3
*No, most of the time I have coped quite well*	116	62.4
*No, I have been coping as well as ever*	43	23.1
***7. I have been so unhappy that I have had difficulty sleeping***		
*Yes, most of the time*	52	28.0
*Yes, sometimes*	78	41.9
*Not very often*	25	13.4
*No, not at all*	31	16.7
***8. I have felt sad or miserable***		
*Yes, most of the time*	2	1.1
*Yes, sometimes*	22	11.8
*Not very often*	83	44.6
*No, not at all*	79	42.5
***9. I have been so unhappy that I have been crying***		
*Yes, most of the time*	60	32.3
*Yes, quite often*	54	29.0
*Only occasionally*	39	21.0
*No, never*	33	17.7
***10. The thought of harming myself has occurred to me***		
*Yes, quite often*	0	0
*Sometimes*	2	1.1
*Hardly ever*	13	7.0
*Never*	186	91.9

**Fig. 1:**
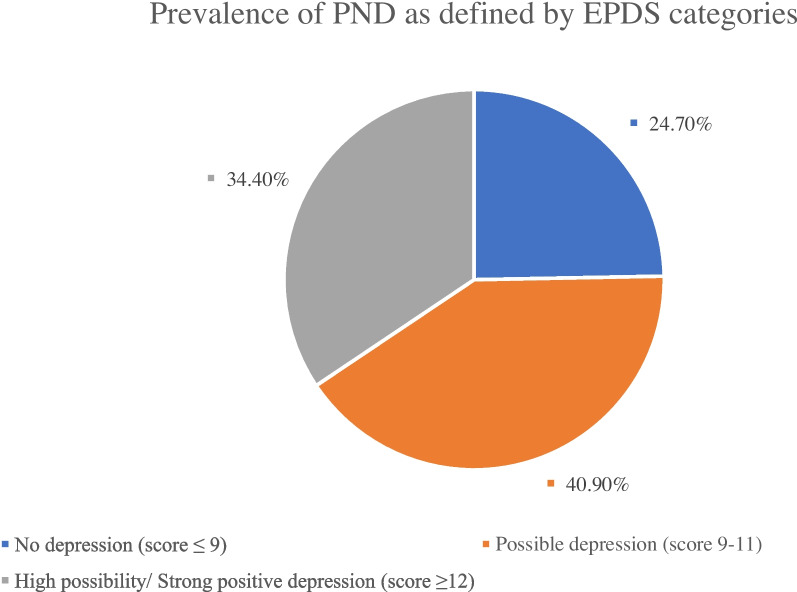
Depression categories defined by EPDS scores

Scores on the EPDS scale were used to classify the recruited women into depression groups, as shown in Fig. 1. A total of 24.7% had no depression (score ≤ 9), 40.9% had possible depression (score 9–11) and 34.4% had a high depression possibility or strong positive depression (score ≥ 12).

### Predictors of perinatal depression

Table 3 presents the associations between PND and sociodemographic and clinical factors among women with GDM.

**Table 3:** Depression Categories by Predictors (chi-square test)

*Variables*	*No Depression* *(score* ≤ *8)*	*Possible depression (score (score 9–11*)	*High possibility/Strong positive depression (score* ≥ *12)*	*Chi-Square value*	*p value*
***GDM***					0.211
*Yes*	27.5%	43.1%	29.6%		
*No*	20.8%	37.7%	41.6%	5.680^a^	
***Age***					0.013*
*18–25*	19.0%	38.1%	42.9%	12.602^a^	
*26–35*	20.0%	41.9%	38.1%		
*36–45*	43.6%	41.0%	15.4%		
***Nationality***				7.842^a^	0.020*
*Emirati*	21.3%	40.0%	38.7%		
*Non-Emirati*	38.9%	44.4%	16.7%		
***BMI***					0.031*
*Underweight/Normal*	17.5%	32.5%	50.0%	10.646^a^	
*Overweight*	24.2%	53.0%	22.7%		
*Obese*	28.7%	35.0%	36.3%		
***Planned pregnancy***				4.236^a^	0.120
*Yes*	20.8%	44.6%	34.6%		
*No*	33.9%	32.1%	33.9%		
***Personal History of depression***					0.004*
*Yes*	3.6%	39.3%	57.1%	10.939^a^	
*No*	28.5%	41.1%	30.4%		
***Previous history of mood disorder***				5.275^a^	0.072
*Yes*	11.1%	37.0%	51.9%		
*No*	27.9%	41.5%	31.4%		

^*^The *P* value < 0.05 indicates statistical significance

Age was significantly and negatively associated with the occurrence of PND among pregnant women with GDM. A high possibility/strong positivity of depression was reported in 42.9% of women in the age group of 18–25, 38.1% in women of age 26–35, and 15.4% in those of age 36–45 years (chi-square = 12.602, df = 4, *p* value = 0.013).

Nationality was another factor that was significantly associated with PND, where a high possibility/strong positive depression was reported by 38.7% of Emirati women compared to 16.7% among non-Emirati women (chi-square = 7.842, df = 2, *p* value = 0.020).

PND was significantly associated with obesity. The study results showed that a high possibility/strong positive depression was found in 50.0% of women in the underweight/normal BMI group, 22.7% in overweight women, and 36.3% in obese women (chi-square = 10.646, df = 4, *p* value = 0.031).

A history of depression was a significant correlate of PND in the current pregnancy. A high possibility/strong positive depression was reported by 57.1% of women with a history of depression compared to 30.4% of women with no history (chi-square = 10.939, df = 2, *p* value = 0.004).

Pregnancy planning and past medical history of mood disorders were found to be insignificantly associated with PND among GDM women. A proportion of 34.6% of women who had planned pregnancies showed a high possibility/strong positive depression, compared to 33.9% of women with unplanned pregnancies (Chi-square = 4.236, df = 2, *p* value = 0.120). Past medical history of mood disorders showed borderline significance when correlated with PND. Highly possible depression/strong positive depression was found in 51.9% of women reporting a past medical history of mood disorders compared to 31.4% in those with no history of mood disorders (chi-square = 5.275, df = 2, *p* value = 0.072).

### Regression analysis

Multivariate binary logistic regression was used to identify significant predictors of “High possibility/Strong positive depression”. The regression model included GDM, age, nationality, BMI, history of depression, and history of mood disorders as input variables (Table 4). The model was adequate in predicting the outcome variable (Hosmer and Lemeshow test Chi-square = 4.935, *p* value = 0.765; Omnibus test Chi-square = 29.869, *p* value < 0.001). The regression model was able to predict between 14.8% and 20.5% of the variance in the outcome variable.

**Table 4:** Binary logistic regression model predicting high “possibility/strong possible depression” in pregnant women

Variables	***B***	***S.E***	***p value***	Odds Ratio		
				***Value***	95% Confidence Interval	
					Lower	Upper
GDM						
No	0.454	0.357	0.204	1.574	0.781	3.172
Yes				1		
Age						
18 – 25	1.110	0.588	0.059	3.035	0.959	9.609
26 – 35	1.094	0.519	0.035*	2.986	1.079	8.259
36 – 45				1		
Nationality						
Non-Emirati				1		
Emirati	0.993	0.512	0.053	2.699	0.989	7.366
Past History of Depression						
No				1		
Yes	1.001	0.478	0.036*	2.721	1.067	6.941
Previous History of Mood Disorders						
No				1		
Yes	0.429	0.482	0.373	1.536	0.597	3.951
BMI						
Underweight/Normal				1		
Overweight	-1.094	0.469	0.020*	0.335	0.133	0.840
Obese	-0.214	0.452	0.636	0.807	0.333	1.959

^*^The *P* value < 0.05 indicates statistical significance

Three out of the six input variables were significant predictors of “High possibility/strong positive depression”. Women aged 26 to 35 years were 2.986 times more likely to report a high possibility/strong positive depression (95% confidence interval 1.079—8.259; *p* value = 0.035). Women with a history of depression were 2.721 times more likely to have a high possibility/strong positive depression (95% confidence interval 1.067 – 6.941; *p* value = 0.036). Moreover, BMI was found to be a significant negative predictor of the outcome. Women in the overweight group were 2.985 times less likely to report a high possibility/strong positive depression than women with a normal BMI (95% confidence interval 1.190 – 7.519; *p* value = 0.020). Other input variables, including GDM, nationality, and previous history of mood disorders, were nonsignificant predictors of high possibility/strong positive depression (Table 4).

## Discussion

In a study conducted among Emirati women in 2020, as reported in the latest edition of the International Diabetes Federation, the prevalence of GDM demonstrated a significant increase. The incidence rose from 7.9% to 37.7%, representing a substantial statistical increase of 29.8% [15]. Similarly, within the scope of this study, where 81.2% of Emirati participants were considered, an alarming 58.6% of women were found to have GDM, indicating a notable 50% increase in this estimate.

Revising factors correlation literature view in regards to predicted age factor study analysis “among the age group of (26–35 years) 41.9% of women had High possibility\High positive depression (score ≥ 12)”; this finding aligns with the results of the "LINDA-Brazil study," which conducted a comprehensive investigation into depression. Specifically, this study concluded that 50% of women aged between 30 and 39 years not only displayed depressive symptoms but also reported a concerning inclination toward self-harm [16].

Anthropometric analysis reported that 43% of participants were obese, which is considered a dependent risk factor for GDM among pregnant women. In a study assessing GDM combined risk factors among overweight or obese women, a significant association was found (OR = 1.44, 95% CI = 1.04–1.81, *p* value 0.02), supporting the statement that obesity is a dependent risk factor for GDM [17]. Similarly, reviewing the literature evaluating factors pertaining to sociodemographic variables and their correlation with both depression and GDM in a cross-sectional analytical study (2018) in India among 347 women above twenty-four weeks gestational age, the Clinically Useful Depression Outcome Scale (CUDOS) questionnaire was applied and concluded that women with GDM and a BMI of 27.14 and above have a further increased risk of depression susceptibility [11].

Depression was found to be related to a past medical history of depression in this study, where 57.1% of women who had depressive symptoms had a high depression possibility/strong positive depression scored ≥ 12. Similarly, a large descriptive cross-sectional study examining subsequent antenatal mental disorders (depression, anxiety, and stress symptomology) among women with GDM found that pregnant women with GDM had the highest prevalence of anxiety symptoms (39.9%), followed by depressive symptoms (12.5%) and stress symptoms (10.6%) [16]. In addition, a recent study published in 2021 showed that depression during pregnancy (OR = 4.25; 95% CI, 3.28- 5.50; *P* < 0.001) was associated with a high prevalence of PND [5]. Thus, depression is the second most prevalent mental disorder found among women with GDM.

In agreement with the present findings, a study by LINDA-Brazil conducted on 820 women with GDM investigated depressive symptom frequency and severity and their relationship with sociodemographic characteristics and showed that 50% were living with their partners, 88% had low socioeconomic status and 39% had one to two minimum family income remunerations [16]. Similarly, our study has revealed that self-accomplishment, marital satisfaction and living arrangements contribute significantly to the development of postpartum depression or mood disorders during pregnancy.

Among pregnant women diagnosed with GDM, the study findings reveal a notable association with depression. Specifically, 43.1% of these women scored within the range indicating possible depression (scores 9–11), while a significant 29.6% displayed a high likelihood of experiencing severe depression, marked by scores equal to or exceeding 12. It is noteworthy that the statistical analysis yielded an estimated *p* value of 0.211.

While there is a discernible trend toward an increased risk of PND among women with GDM, it is crucial to note that this trend did not reach statistical significance in the analysis.

However, in a case‒control study examining the relationship between gestational diabetes and a positive depression screen, a total of 315 pregnant women with GDM scored positive 33.7% (104) for depression (EPDS overall scores below the cutoff levels of 13) compared to the percentage of pregnant women without gestational diabetes mellitus (no GDM), who score positive 6.2% (211) for depression (EPDS overall scores below the cutoff levels of 13) with an estimated (*p* < 0.001) odds ratio of (6.7) and (95%) confidence interval (3.3–13.6), revealing an interestingly high percentage of depression among GDM pregnant women compared to non-GDM women [18].

Intriguingly, the present findings propose that GDM was not a very strong predictor of depression, which may be due to the following limitations: (i) short observation period of the pregnant and postnatal women who were diagnosed with GDM as the questionnaires were cross-sectional and candidates were not followed, (ii) having a previous personal history of depression (which is a strong predictor of having subsequent PND) masked the effect of GDM. Therefore, further studies with longer observational periods might reveal the relationship between GDM and perinatal depression.

In addition, this study has some limitations due to using a self-reporting questionnaire which makes it subject to response bias and the small sample size as the study was conducted in one center. Another limitation is that not all subjects from the population had the opportunity to participate in the survey, which therefore leads to nonresponse bias that affects the reliability of the survey’s results. The questionnaire was completed by pregnant and postnatal women during an outpatient visit, which was usually surrounded by their partners, making it subject to introspective bias. The representativeness of the sample was not guaranteed due to the use of the snowball sampling method, where there is little control in recruiting participants.

## Conclusions

The frequency of PND among Emirati women with GDM indicates a prognostic statistical increase of 50% in this study. The study findings propose that age, personal history of depression, and BMI are very strong predictors of perinatal depression during pregnancy. Our results suggest the need for early screening of perinatal depression, especially in more vulnerable populations. Although GDM was not positively correlated with PND in the results of this study, further studies with longer observational periods might reveal the relationship between GDM and PND.

## Data Availability

The datasets used and/or analyzed during the current study are available from the corresponding author on reasonable request.

## Abbreviations

BMI: Body Mass Index
CI: Confidence Interval
CUDOS: Clinically Useful Depression Outcome Scale
T2DM: Type 2 Diabetes Mellitus
EPDS: Edinburgh Postnatal Depression Scale
GDM: Gestational Diabetes Mellitus
GTT: Glucose Tolerance Test
NGT: Normal Glucose Test
Non-GDM: Non-gestational diabetes mellitus
OGTT: Oral glucose tolerance test
OR: Odds Ratio
PND: Perinatal Depression
SD: Standard Deviations
UAE: United Arab Emirates
UHS: University Hospital Sharjah
UOS: University Of Sharjah

## References

[1] Committee American Diabetes Association Professional Practice (2021). 2. Classification and Diagnosis of Diabetes: Standards of Medical Care in Diabetes—2022. Diabetes Care..

[2] Gazal M, Motta LS, Wiener CD, Fernandes JC, Quevedo LÁ, Jansen K (2012). Brain-derived neurotrophic factor in post-partum depressive mothers. Neurochem Res.

[3] Alkhatatbeh MJ, Abdalqader NA, Alqudah MA (2019). Impaired awareness of hypoglycemia in insulin-treated type 2 diabetes mellitus. Curr Diabetes Rev.

[4] Goldenberg RL, McClure EM, Harrison MS, Miodovnik M (2016). Diabetes during pregnancy in low-and middle-income countries. Am J Perinatol.

[5] Peng S, Lai X, Du Y, Meng L, Gan Y, Zhang X (2021). Prevalence and risk factors for postpartum depression in China: A hospital-based cross-sectional study. J Affect Disord.

[6] Gaynes BN, Gavin N, Meltzer-Brody S, Lohr KN, Swinson T, Gartlehner G (2005). Perinatal depression: Prevalence, screening accuracy, and screening outcomes: Summary. AHRQ evidence report summaries.

[7] Gavin NI, Gaynes BN, Lohr KN, Meltzer-Brody S, Gartlehner G, Swinson T (2005). Perinatal depression: a systematic review of prevalence and incidence. Obstetr Gynecol..

[8] Wei L (2019). Factors associated with depression, anxiety, stress and adverse neonatal outcomes among gestational diabetes mellitus patients in two hospitals in the klang valley, malaysia.

[9] Simas TAM, Whelan A, Byatt N. Postpartum depression—new screening recommendations and treatments. JAMA. 2023;330(23):2295–6. 10.1001/jama.2023.21311. 10.1001/jama.2023.21311 38010647

[10] Depression P, Causes AT (2015). Heterogeneity of postpartum depression: a latent class analysis. Lancet Psychiatry.

[11] Saha A. Understanding the prevalence of depression in pregnant women with gestational diabetes mellitus and its association with metabolic profile. Education. 6323:0.

[12] Silverman ME, Reichenberg A, Savitz DA, Cnattingius S, Lichtenstein P, Hultman CM (2017). The risk factors for postpartum depression: A population-based study. Depress Anxiety.

[13] Hinkle SN, Buck Louis GM, Rawal S, Zhu Y, Albert PS, Zhang C (2016). A longitudinal study of depression and gestational diabetes in pregnancy and the postpartum period. Diabetologia.

[14] Cox JL, Holden JM, Sagovsky R (1987). Detection of postnatal depression: Development of the 10-item Edinburgh Postnatal Depression Scale. Br J Psychiatry.

[15] Agarwal MM (2020). Gestational diabetes in the Arab gulf countries: Sitting on a land-mine. Int J Environ Res Public Health.

[16] Dame P, Cherubini K, Goveia P, Pena G, Galliano L, Facanha C, Nunes MA. Depressive symptoms in women with gestational diabetes mellitus: the LINDA-Brazil study. J Diabetes Res. 2017;2017:7341893. 10.1155/2017/7341893. 10.1155/2017/7341893 PMC5480043 28685151

[17] Yong HY, Mohd Shariff Z, Mohd Yusof BN, Rejali Z, Tee YYS, Bindels J (2020). Independent and combined effects of age, body mass index, and gestational weight gain on the risk of gestational diabetes mellitus. Sci Rep.

[18] Ozelle OH (2019). Relationship between gestational diabetes and a positive depression screen.

